# Key parameters and sensitivity analysis of lower limb muscle strength in young men with different gait patterns

**DOI:** 10.1371/journal.pone.0318693

**Published:** 2025-03-19

**Authors:** Yifei Wang, Jianbo Sun, Xuesong Guo, Delong Dong, Chuankai Luan, Xiaolin Wang

**Affiliations:** 1 College of Sports Sciences, Qufu Normal University, Jining, China; 2 Exercise and Sports Science Programme, School of Health Sciences, Universiti Sains Malaysia, Kota Bharu, Kelantan, Malaysia; 3 Department of Physical Education, Ludong University, Yantai, China; 4 Department of Sports Studies, Faculty of Educational Studies, University Putra Malaysia, Selangor, Malaysia; Ningbo University, CHINA

## Abstract

**Objective:**

To explore the functional characteristics and principal component differences of electromyography in different phases of the gait cycle, to provide key parameters for identifying a complete gait, and to provide a reference for joint moment solving in the lower limb.

**Methods:**

Twenty young men were selected to measure the natural gait EMG of 14 muscles of the lower limb using VICON and NORAXON devices. Gait was classified into two categories according to the Niyogi S A classification, integral EMG differences were compared, and principal component analysis was performed on the differing muscles to calculate Cohen’s d values for significant differences and ΔIEMG values for non-significant differences.

**Results:**

(1) Significant differences existed in the integral EMG of the left semitendinosus, right semitendinosus, right biceps femoris, and left gastrocnemius muscles, both lateral and medial. (2) Principal component analysis showed significant differences in the left semitendinosus for principal component five (P < 0.1, ES = 1.40); right biceps femoris for principal component three (ES = 0.63, 10%-30%); and left gastrocnemius medial for principal component four (P < 0.05, ES = 1.81, 40%-60%). The ΔIEMG% of the right semitendinosus principal components I-IV were 97.96%, 92.24%, 87.26%, and 75.08%, respectively; and the ΔIEMG% of the left gastrocnemius medial principal components I-IV were 90.95%, 75.08%, 96.37%, and 85.39%, respectively.

**Conclusion:**

(1) Left semitendinosus, right semitendinosus, right biceps femoris, and left gastrocnemius can be used as the main muscles for gait recognition. (2) The left semitendinosus principal component V, the right biceps femoris principal component III, and the left gastrocnemius medial principal component IV are sensitive indicators for gait stage classification.

## Introduction

Walking is widely recommended by healthcare professionals as a simple and effective form of exercise, as it helps to prevent joint, heart and metabolic diseases, and has a particularly positive role in the risk management of diabetes. Therefore, enhancing the efficiency of physical function recovery through gait analysis has become the core goal of a variety of rehabilitation training programmes. The identification of gait EMG parameters can not only reveal gait characteristics and patterns and help correct abnormal gait, but also be used in human-computer interaction intelligence research, which is an important direction in the field of human movement biomechanics [1–2]. Ravera et al. stated that EMG is an important parameter for dynamic muscle strength assessment in gait analysis [3]. The joint moment solution method proposed by Xunfeng Yin improves the accuracy of multigait estimation of lower limb joint moments in normal gait [4]. On the other hand, investigated the difference between the gait of prosthesis users and normal people through a motion capture system, which further provided an important basis for rehabilitation training [5].

Currently, academic measurements of human gait include gait indices, myoelectric fusion modelling and inverse dynamics solving. Three-dimensional geometric and kinematic techniques are commonly used for gait assessment. Scholars have investigated the relationship between weight-bearing and muscle activity, changes in gait angle at different running speeds, analysed the effect of gait speed on gait in normally developing children, and tested the relationship between walking speed and gait, laying the groundwork for the study of the temporal phase of gait and key variables [6–10].

Walking, although a simple form of alternating leg movement, involves a complex collaboration of cognition, attention, muscle strength, and motor control [11]. Movement disorders lead to increased gait variability and asymmetry, which in turn affects walking efficiency and increases energy expenditure. Gastrocnemius and piriformis muscles, among others, play a role in walking by providing power to the supporting leg, while hip and knee joint movement is facilitated by the collaboration of hamstrings and quadriceps to propel the body forward and mitigate impact during landing [12]. However, despite the important role these muscle groups play in gait, it is not yet possible to ignore the contribution of other muscles.

Research on the identification of gait EMG parameters in different populations is developing intensively, but studies on sensitive parameters and key time phases for normal gait are still relatively lacking. Lower limb gait surface EMG data is a continuous and complete process, and the sequential nature of the data may be neglected using traditional analysis methods. Therefore, this study chose to use Functional Data Analysis (FDA), a method proposed by Ramsay, President of the Statistical Society of Canada, which is based on the principle of treating the observed data as a whole and fitting it to a continuous curve in order to provide in-depth analyses of the whole curve [13]. Functional data analysis has been widely used in the fields of movement development, movement technique analysis, sports injury diagnosis, and evaluation of rehabilitation effects [14–17]. Therefore, in this study, data from the gastrocnemius, hamstring and quadriceps muscles of young males were collected by surface electromyography with the aim of: i) identifying the different gait types and their electromyographic characteristics; and ii) revealing the key identifying parameters of gait characteristics and the mechanisms that influence them. Since the performance of ideal gait is closely related to age and physical characteristics, young males aged 22-24 years were selected as the subjects of this study, with the expectation that it would provide reference and guidance for gait optimisation as well as the measurement of joint moments of the lower limbs in this age group.

## Research object and method

### Test objects

In this study, repeated measures analysis of variance (ANOVA) was conducted using G-Power software to calculate the sample size. An effect size (d) of 0.3, a significance level (A) of 0.05, and a power of 0.80 were chosen to test six measurements at a time. Analyses determined that a minimum of 11 participants was required. The recruitment period for participants is from March 5, 2022 to April 5, 2022. Eleven young healthy males were selected as test subjects, and the age inclusion criteria were based on the youth age segments (10-24 years old) determined by the World Health Organisation, and the test subjects in this study were selected on the basis of the following criteria: (1) age of 22-24 years old; (2) physical and mental health, with no history of infectious or genetic diseases, and no drug dependence; (3) normal movement of all body joints; (5) no special groups (super-fat, flat feet, etc.), no sports injuries in the last six months; (6) The data obtained in this experiment are valid data, and all subjects have filled in the written informed consent in advance. Participants were informed about the purpose, procedures, potential risks, and benefits of the study. This study was approved by the Academic Ethics Committee of the University (approval number: LDU-IRB202106011). Finally, 11 subjects were included (see Table 1 for specific information).

**Table 1:** List of basic information of test subjects.

Genders	N	Age/years	Height/cm	Weight/kg
Males	11	23.16 ± 0.55	177.13 ± 4.88	73.73 ± 8.33

### Test method

In this study, VICON infrared 3D motion capture system, infrared high-definition camera (12 camera points, 120Hz) and Noraxon Ultium surface electromyography acquisition and analysis system (sampling frequency: 2000Hz) were selected for data acquisition. In order to ensure the consistency and synchronization of data acquisition, the VICON system and the Noraxon system are time-synchronized through the Nexus 3.12 software, ensuring that the motion capture data and the EMG data are analyzed on the same timeline. Other auxiliary equipment includes shaver, 75% medical alcohol, electromyographic adhesive, elastic bandage, tape, etc.

Through the VICON 3D motion capture system and the Noraxon Ultium32 wireless surface myoelectrometer combined with the computer software Nexus 3.12, we took a panoramic shot of the natural gait of 20 young men, captured the motion trajectory, and obtained the three-dimensional motion coordinates of the reflection spot of the test movement and other biomechanical parameters. Surface EMG data were collected using 16 Noraxon Ultium32 conduction wireless EMG meters with a sampling frequency of 2000Hz.

Before the test, the researchers prepared the subjects with disposable myoelectric patches, medical cotton balls, computers, alcohol wipes, paper towels, tape, razors and subjects’ clothing. 16 reflective tracking points were fixed on both legs of the subjects, which were located on the anterior superior iliac spine, posterior superior iliac spine, middle thigh, lateral knee joint, middle calf, lateral ankle joint, heel and toe respectively. Before the test, the subjects were informed of all the test procedures, and their height and weight were measured and recorded. Subsequently, the local skin is wiped with alcohol wipes and dried to ensure the quality of the EMG acquisition.

**Fig 1:**
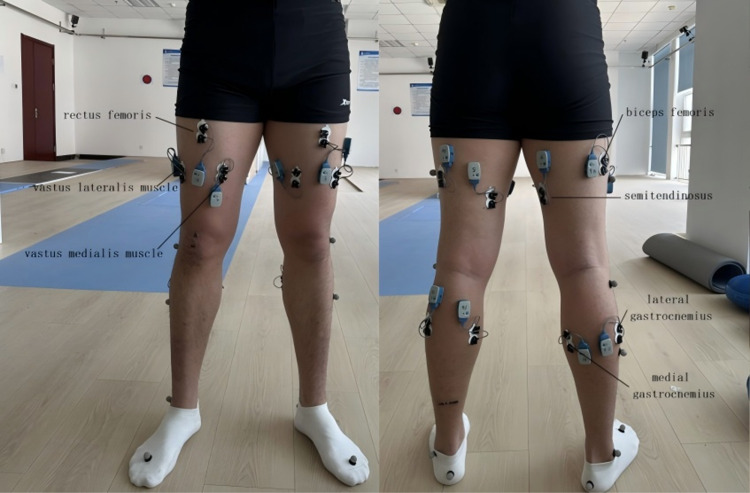
Schematic of subject preparation.

**Fig 2:**
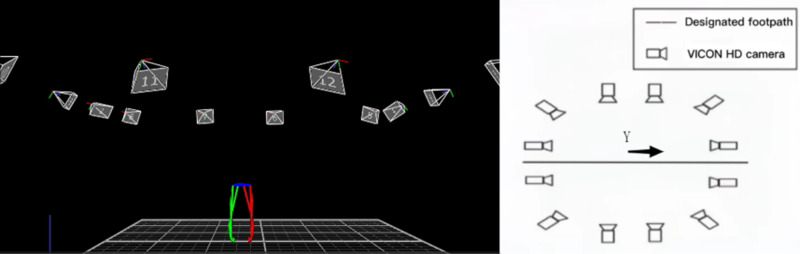
Lower limb model and test area.

**Fig 3:**
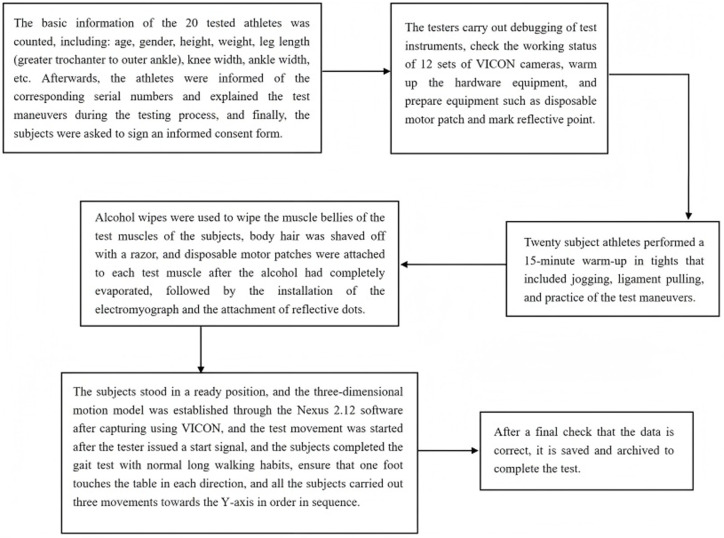
Test flow chart.

During the test, a professional laboratory technician places electromyometer electrodes on the subject in a predetermined position, covering the muscular abdomen of the rectus femoris, lateralis femoris, medialis femoris, semitendinosus, biceps femoris, lateral head of gastrocnemius and medial head of gastrocnemius. The subject then stood on a force table and a lower limb mannequin was built. At the beginning of the formal test, the subject walks without stopping through the designated test area with a natural gait under the guidance of a professional (Figs 1–3).

### Data processing

#### Division of gait cycle.

In order to clearly analyze the EMG characteristics of lower limb muscles, this study used VICON Nexus 3.12 software to capture the gait data of subjects, and used infrared cameras and markers to track the movement trajectory of lower limbs. The Nexus 3.12 software not only provides accurate 3D motion data, but can also export video files that show the subject’s movements at various stages of the gait cycle. Through the software, we obtain the three-dimensional coordinates and motion trajectories of each time point, and can generate video files related to the gait period.

The exported gait data and video files were then further analyzed and calibrated using Adobe After Effects 2021 software. The specific operation is to identify key moments in different stages of the gait cycle (such as load bearing period, single support period, pre-swing period and swing period) through reflective markers in the video. For example, in the load-bearing period, the start and end of the load-bearing period are determined by marking the moment when the right foot follows the ground and the moment when the left toe lifts off the ground; In the swing period, the start and end point of the swing period is determined by marking the moment when the right toe is off the ground and the right foot is following the ground.

Using Adobe After Effects’ Timeline tool, we were able to calculate the duration of each gait stage. The results of the time axis analysis for each gait cycle are used to calculate the specific duration of each stage. Through the analysis of several gait cycles, we obtained the time ratio of different gait types in the support phase and the swing phase, so as to further provide a basis for the analysis of EMG data.

**Fig 4:**
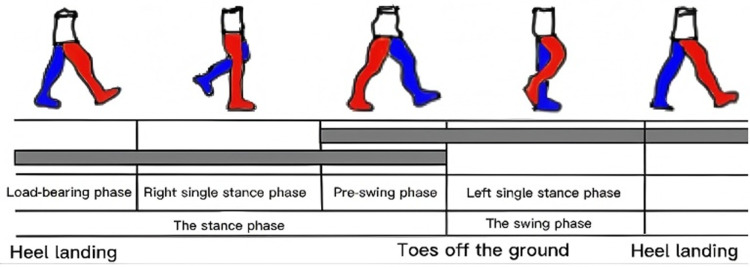
Division of the gait cycle.

In gait analysis, the gait cycle is divided into support stage and swing stage, and the support stage is subdivided into three sub-stages: the first double support stage (load-bearing stage), the single support stage and the second double support stage (pre-swing stage). Taking the right side as an example, the load-bearing phase refers to the moment when the right foot follows the ground until the left toe is off the ground; The single support stage is the moment when the left toe is off the ground and the left foot is following the ground. The preswing phase begins with the left foot following the ground and ends with the right toe lifting off the ground. The swing phase starts with the right toe off the ground and ends with the right foot following (Fig 4) [18]. The duration of each stage was derived from the above calibration and timeline analysis and used for subsequent EMG analysis.

### Qualitative analysis of the developmental sequence of natural walking lower limb movement components

Referring to the Niyogi S A study, it was suggested that for a normal gait cycle, the support phase accounts for approximately 60%-62% of the entire gait cycle and the swing phase accounts for approximately 38%-40% of the entire gait cycle [19]. Based on this criterion, the proportions of the support phase and swing phase were counted (Table 2), and the lower limb movements in walking were classified into 2 types. Type I was the support phase accounting for approximately 60%-62% of the entire gait cycle and the swing phase accounting for approximately 38%-40% of the entire gait cycle (Type I gait); those outside the range of the normal interval were classified as Type II gait.

**Table 2:** Support phase and swing phase times and approximate percentages of the total gait cycle.

Encoding (People)	Gait Type	Stance phase time(s)	Swing phase time (s)	Total time(s)	Stance phase ratio (%)	Swing phase ratio (%)
One	Ⅰ	0.72	0.47	1.19	60.5	39.5
Two	Ⅰ	0.65	0.42	1.07	60.7	39.3
Three	Ⅱ	0.63	0.44	1.07	58.9	41.1
Four	Ⅰ	0.74	0.46	1.20	61.7	38.3
Five	Ⅱ	0.69	0.50	1.19	58.0	42.0
Six	Ⅱ	0.64	0.47	1.11	57.7	42.3
Seven	Ⅱ	0.75	0.42	1.17	64.1	35.9
Eight	Ⅱ	0.7	0.42	1.12	62.5	37.5
Nine	Ⅱ	0.75	0.43	1.18	63.6	36.4
Ten	Ⅰ	0.71	0.46	1.17	60.7	39.3
Eleven	Ⅱ	0.70	0.48	1.18	59.3	40.7

### EMG data acquisition

EMG data processing uses NORAXON’s own MR3.16 software to report the standard surface EMG, respectively, IEMG, activation order and other data export. Among them, IEMG is the integral of the region encircled between the EMG change curve and the time transverse axis in EMG, which can reflect the intensity of muscle EMG activity at a certain moment and indicate the degree of force exerted by the muscle during the working process, i.e., the greater the value of the integral EMG, the greater the degree of force exerted by the muscle. Therefore, the lower limb muscle force generation characteristics in natural gait can be clearly observed (Equation (1)) [20–21]; the activation sequence can better examine the sequence of muscle force generation in the whole process and demonstrate the force generation characteristics.


$$IEMG=\sum_{i=1}^{n}\left|x_{i}\right|$$ (1)

#### Function-based data analysis.

Function-based data analysis was implemented in matlab2021a using the FDA’s FPCA toolkit programming [13]. Gait is a periodic movement, therefore, a 3rd order Fourier basis was used to fit the lower limb surface EMG time series curves to a function [15], with the smoothing parameter set to e-7. Subsequently, the function was downscaled and decomposed into a number of principal components, with cumulative contribution of 85% and eigenvalues of each principal component greater than 1 [22].

The specific formula is as follows:


∫a(s,t)ξ(t)dt=μξ(s) (2)

$$\omega (t)=\xi (t)\times \sqrt{v}$$ (3)

$$Y_{i}=\int \xi (t)x_{i}(t)dt$$ (4)

The a(s,t) in Equation (2) is the covariance function of the function variable, ξ(t) is the eigenfunction, and μ is the principal component covariance matrix; ɷ(t) in Equation (3) is the principal component weighted weight coefficient function; Yi in Equation (4) is the score of the original function variable xi (t) on each principal component, and the corresponding eigenvalues of each principal component are calculated by the above equation, and then encoded in matlab2021a for the mean ±  weight coefficient values in order to identify the integral EMG of the muscles under different types below.

The specific coding is as follows:

for n = 1:2149

if(MAXelement(n,1)==1)

V1value(:,n)=Resultdata(:,n);

V1=V1value(:,any(V1value,1));

 end

if(MAXelement(n,1)==2)

V2value(:,n)=Resultdata(:,n);

V2=V2value(:,any(V2value,1));

 end

 ...

data1(n,1)= meanValue(n,1)+meanValue(n,1)*MAXValue(n,1);

data2(n,1)= meanValue(n,1)-meanValue(n,1)*MAXValue(n,1);

end

#### Mathematical statistics.

A one-way ANOVA was used to compare and analyse the principal element scores of the lower limb surface EMG time series curves of young men with different gait types respectively, with the significance level set at 0.1, and the follow-up test was performed using the LSD method. On this basis, stepwise discriminant analysis was used to further screen out sensitive indicators that could reflect the gait characteristics of young men by taking the principal element scores of the lower limb surface EMG curves with significant differences screened out in the analysis of variance as the independent variables, and different gait types as the grouping dependent variables. The data analysis process was carried out using SPSS21.0 software. After screening the muscle strength sensitive indicators of gait characteristics, their Cohen’s d effect size (ES) size was calculated, and they were classified as no effect, low effect, medium effect, high effect and great effect according to the thresholds described by Hopkins et al., i.e., ES < 0.20, 0.20 to 0.60, 0.60 to 1.20, 1.20 to 2.0, and ES ≥  2.0 [23]. The ΔIEMG will be calculated for the screened indicators that are not muscle strength sensitive (Equation (5), ɷ is the weighting factor).


$$\Delta IEMG=\frac{\left(M_{iemg}+M_{iemg}\times \omega )-(M_{iemg}-M_{iemg}\times \omega \right)}{M_{iemg}+M_{iemg}\times \omega }$$ (5)

#### Inclusivity in global research.

Additional information regarding the ethical, cultural, and scientific considerations specific to inclusivity in global research is included in the Supporting Information (SX Checklist).

## Results

### Comparison of integral electromyography of lower limb muscles of different gait types

**Fig 5:**
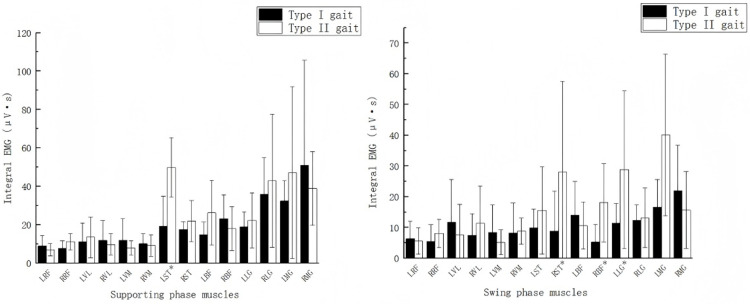
Integral EMG values of lower limb muscles for different gait types.

In order to compare the integral EMG of the lower limb muscles of different gait types, one cycle of the natural walking lower limb movement was divided into the support phase and swing phase, and the integral EMG indexes of the two phases were compared respectively, so as to reflect the participation of each muscle in force generation of the lower limb movement of different gait types. Fig 5 shows the results of the integrated EMG values of the 14 muscles in the support phase and swing phase of the movement. The results showed that there was a difference in gait type of the left semitendinosus in the support phase. The left semitendinosus discharged 19.24 μV·s in the first type of movement and 49.78 μV·s in the second type of movement.In the swing phase, the right semitendinosus, right biceps femoris, left lateral gastrocnemius, and left medial gastrocnemius differed more in the two gait types. The right semitendinosus discharged 8.89 μV·s, the right biceps femoris discharged 5.25 μV·s, the lateral left gastrocnemius 11.4 μV·s, and the medial left gastrocnemius 16.62 μV·s in the Type I gait type. In contrast, the right semitendinosus discharged 28.11 μV·s, the right biceps femoris 18.06 μV·s, the lateral left gastrocnemius 28.85 μV·s, and the medial left gastrocnemius 40.14 μV·s in the class II gait type. This predicts that the left semitendinosus, right semitendinosus, right biceps femoris, left lateral gastrocnemius, and left medial gastrocnemius could serve as the primary identifying muscles for different gait types.

### Functional data analysis of EMG time-series curves of major identified muscles in the lower limb for different gait types

#### Functional data analysis of left semitendinosus integral EMG time-series curve.

**Fig 6:**
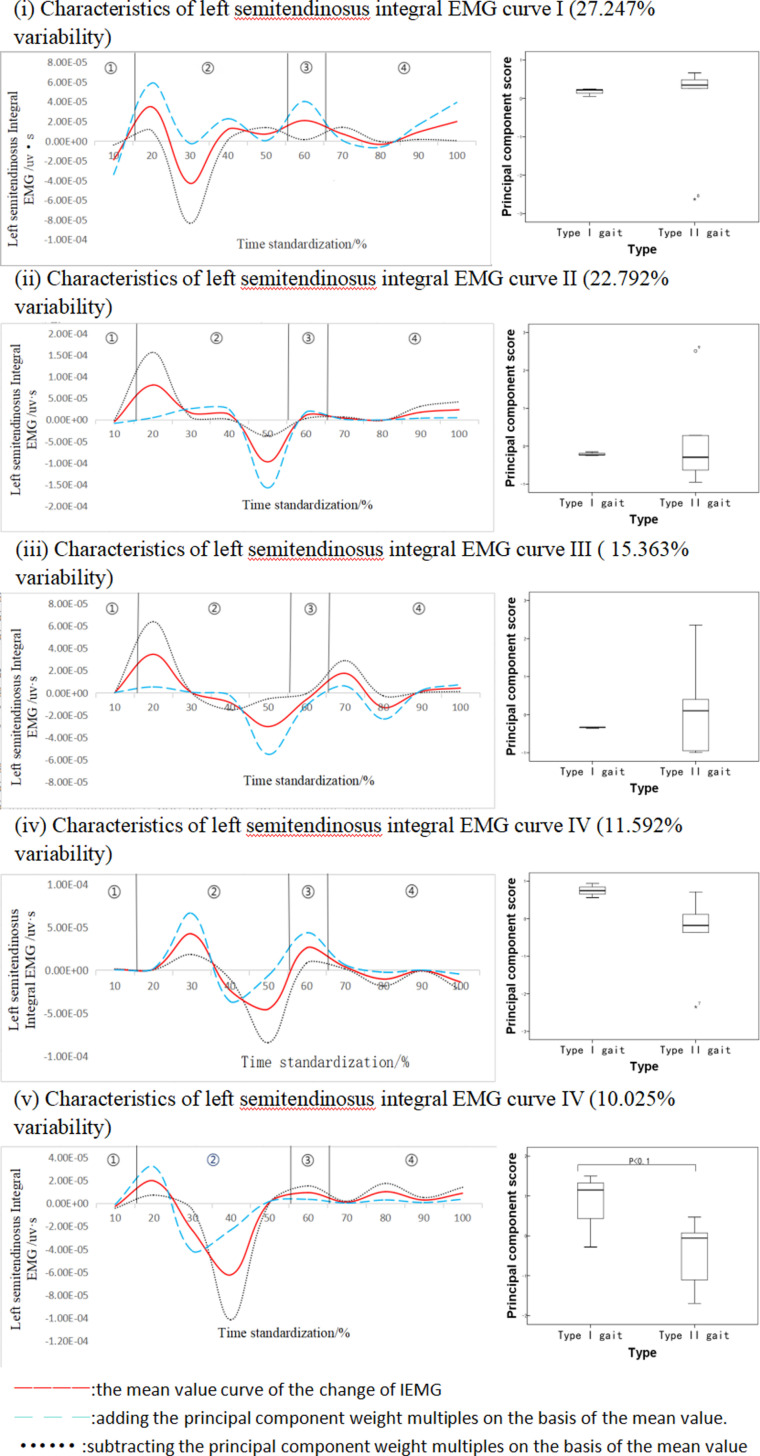
Mean±  weight coefficients of left semitendinosus integral EMG and principal component scores at different stages of movement development.

Fig 6 shows the results of principal component analysis of the integrated EMG time series curve of the left semitendinosus muscle during walking. This integral EMG time series curve can be reduced into five principal components with eigenvalues of 521.059, 435.863, 293.794, 221.680, and 191.713, which express 27.247%, 22.792%, 15.363%, 11.592%, and 10.025% of variability, respectively, with a cumulative contribution rate of 87.019%. Principal component one is the component that contributes the most to the left semitendinosus, although it has the greatest degree of influence on the left semitendinosus and there are multiple fluctuating intervals of variability, the difference in its principal component scores is not significant, indicating that there is no identification of semitendinosus of different gait types at a given time phase from this principal component, and there are basically 2-3 fluctuating intervals for each of the other principal components. Only the fifth principal component has a significant difference, the left semitendinosus integral EMG principal component five-variable fluctuation is concentrated in the second wave valley rising section and the second and third wave peak time, from the right single support period, pre-swing period and left single support period of the three time phases, the left semitendinosus is more discriminative for different gait types. The results showed that subjects with Type I gait had higher scores on this principal component than those with Type II gait, and the difference between the two was statistically significant (P < 0.1, Fig 6v), and also, from the time-series function of each principal component, the time intervals in which the variations occurred were mainly in the range of 30%-50%, with ES = 1.40.

#### Functional data analysis of right semitendinosus integral EMG time series curve.

**Fig 7:**
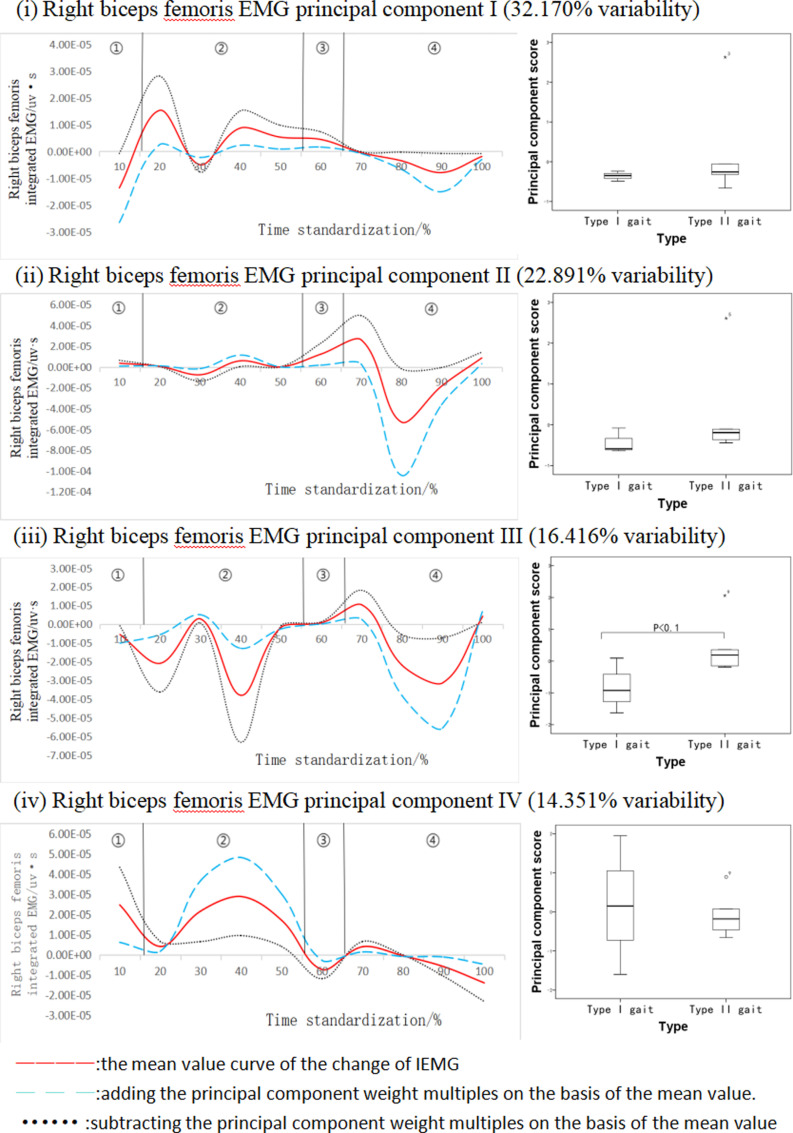
Right semitendinosus integral EMG mean±  weighting coefficients and principal component scores at different stages of movement development.

Fig 7 shows the results of principal component analysis of the integral EMG time series curve of the right semitendinosus muscle during walking. This integral EMG time series curve was downscaled into four principal components with eigenvalues of 643.639, 517.707, 351.387, and 298.178, expressing 31.172%, 25.073%, 17.018%, and 14.441% of variability, respectively, with a cumulative contribution rate of 87.704%. The results showed that all principal component scores of different types of subjects were not statistically significant (Fig 7), but the change curves of each principal component analysis showed that there were two main time intervals of variability (10%-30% and 70%-90% bands), especially the variability characteristics of Principal Component I were more obvious, and the fluctuation of variability of Principal Component III and Principal Component IV significantly occurred in the 70%-90% time interval. Principal component I ΔIEMG% = 97.96%, principal component II ΔIEMG% = 92.24%, principal component III ΔIEMG% = 87.26%, and principal component IV ΔIEMG% = 75.08%, Meanwhile, in terms of principal component scores of principal component IV, the EMG principal component scores of class I gait population were higher than the EMG principal component scores of class II gait population.

#### Functional type data analysis of right biceps femoris muscle integral electromyography time series curve.

**Fig 8:**
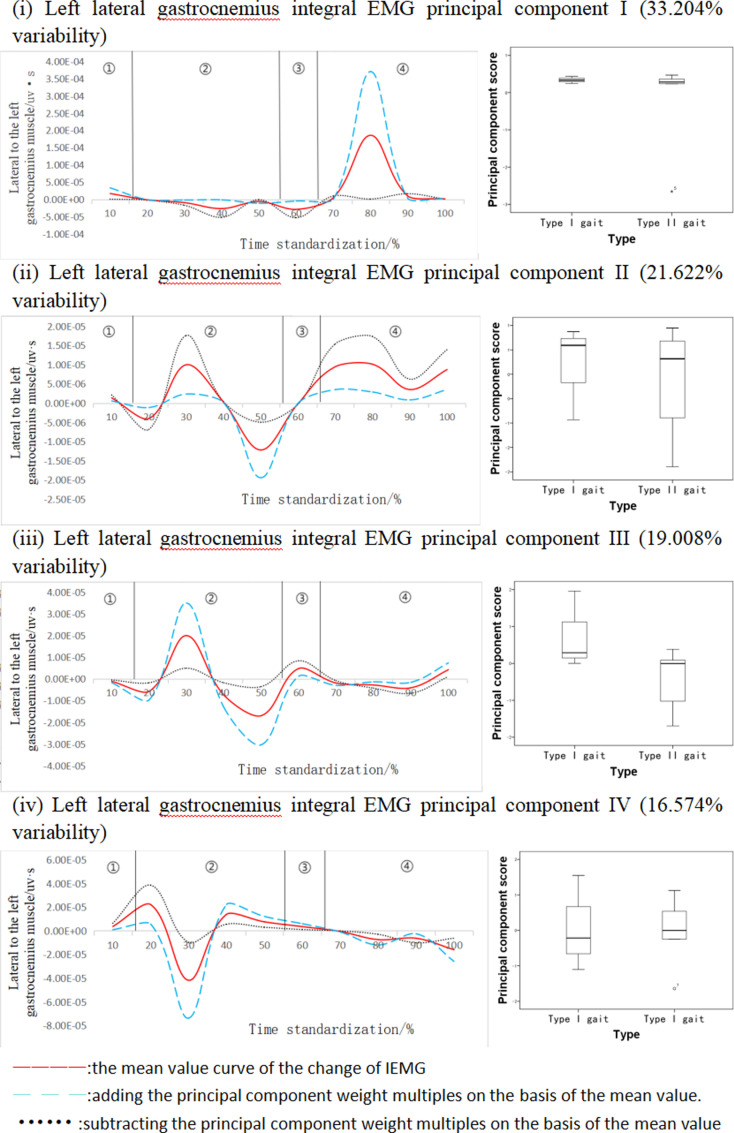
Right biceps femoris muscle integral EMG mean±  weighting coefficients and principal component scores at different stages of movement development.

Fig 8 shows the results of principal component analysis of the right biceps femoris integral EMG time series curve during walking. This integral myoelectric time series curve was downscaled into four principal components with eigenvalues of 505.082, 359.398, 257.738, 225.316, expressing 32.170%, 22.891%, 16.416%, 14.351% of variability respectively, with a cumulative contribution rate of 85.828%. The right biceps femoris integral EMG principal component III showed significant differences in amplitude variability with three variability intervals (10%-30%; 30%-50%; 80%-90%) and the ESs of these three intervals were 0.63, 0.16, and 0.22, respectively, which were concentrated at the peak position, and the scores of this principal component of the subjects with class II gait were higher than those of the subjects with class I gait, and the differences between the two were statistically significant (P < 0.1, Fig 8iii).

#### Data analysis of the left lateral gastrocnemius integral EMG time series curve.

**Fig 9:**
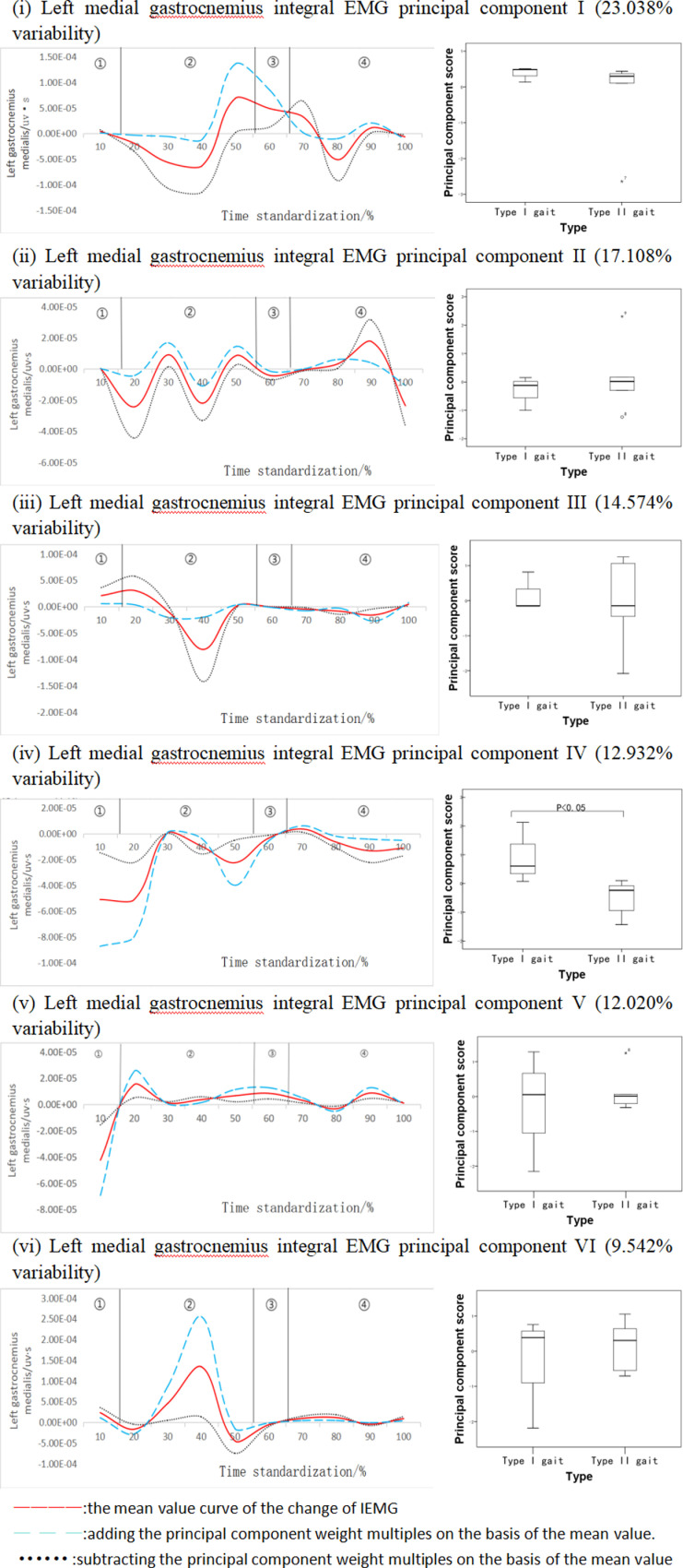
Mean±  weighting coefficients of left gastrocnemius lateral head integral EMG and principal component scores at different stages of movement development.

This integral EMG time series curve was downscaled into four principal components (Fig 9). The eigenvalues were 713.55, 464.66, 408.48, and 365.18, expressing 33.204%, 21.622%, 19.008%, and 16.574% of variability, respectively, with a cumulative contribution of 90.408%. The gait fluctuations showed bimodal or unimodal patterns, with unimodal fluctuations in the 70%-90% or 20%-40% time intervals (Principal Component I and Principal Component IV) and bimodal fluctuations in the 20%-40% and 40%-60% time intervals (Principal Component II and Principal Component III). Principal component one ΔIEMG% =  90.95%, principal component two ΔIEMG% =  75.08%, principal component three ΔIEMG% =  96.37%, and principal component four ΔIEMG% =  85.39%, and all principal component variations occurred in the crest segment position, but different gait types did not show statistically significant differences in each principal component.

#### Data analysis of left medial gastrocnemius integral EMG time series curve.

**Fig 10:**
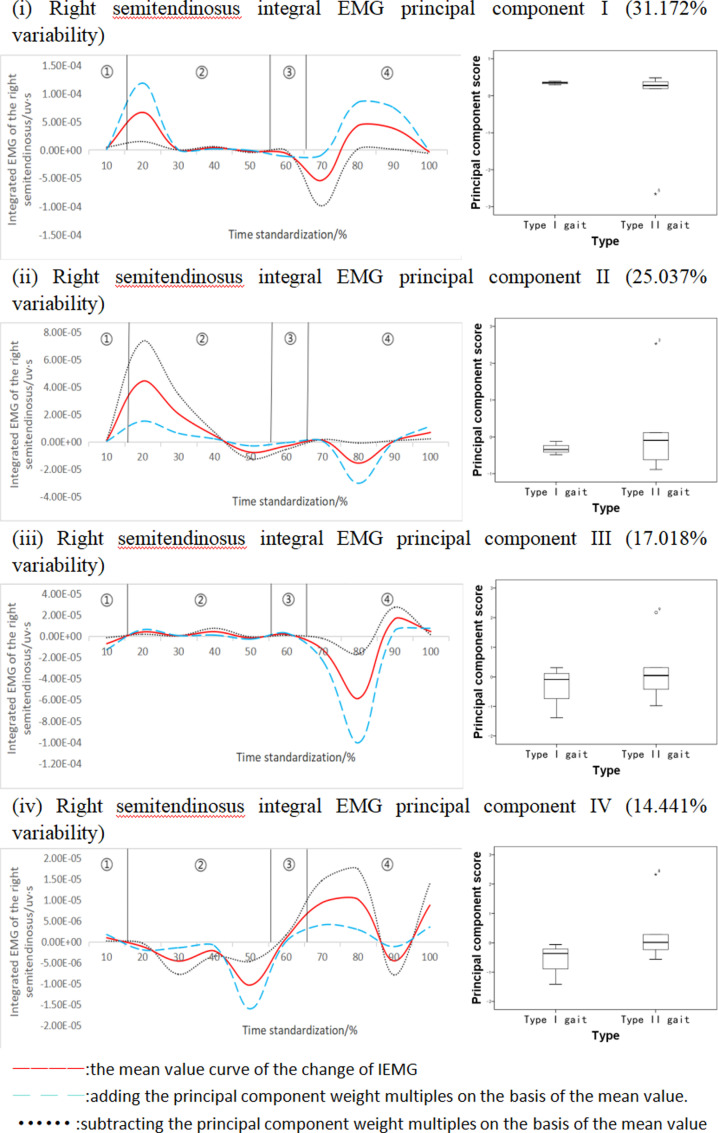
Mean±  weighting coefficients of left medial gastrocnemius head integral EMG and principal component scores at different stages of movement development.

The left medial gastrocnemius head integral EMG time series function curve can be downscaled into six principal components (Fig 10). The eigenvalues were 495.097, 367.659, 313.200, 277.919, 258.308, and 205.057, expressing 23.038%, 17.108%, 14.574%, 12.932%, 12.020%, and 9.542% of the variability, respectively, with a cumulative contribution of 89.215%. The four-amplitude variability of the left gastrocnemius medial head integral EMG principal component showed a three-stage morphology, with the first stage in the 10%-20% time interval, concentrating on the peak position, ES =  1.80; the second stage in the 40%-60% time interval, occurring in the trough rise, ES =  1.81; and the third stage in the 80%-100% time interval, with variability occurring mainly in the trough stage, ES =  0.14, and there were statistically significant differences in the principal component scores of different gait types. At the same time, this principal component score was higher for Type I gait subjects than for Type II gait subjects, and the difference between the two was statistically significant (P < 0.05, Fig 10iv). The time intervals of variation for each of the other principal components were mainly concentrated in the range of 30%-60%, and the scores of the other principal components were not statistically significant for subjects with different gait types.

After stepwise discriminant analysis of the subjects’ lower limb muscle integral EMG principal component scores, three principal components were finally screened out as sensitive indicators of people’s lower limb movement development characteristics during walking, namely, the left semitendinosus principal component IV, the right biceps femoris principal component III, and the left gastrocnemius medial head principal component IV.

## Discussion

The aim of this study was to investigate the activity characteristics of key lower limb muscles (gastrocnemius, hamstrings and quadriceps), especially the sensitivity parameters of these muscle groups in different phases of gait in young males in natural and abnormal gait by using surface electromyography. The results of the study not only provide new data support for gait analysis, but also provide guidance for personalised rehabilitation training.

In the results part of this study, we conducted an in-depth analysis of gait data by principal component analysis and direct analysis. First, we used direct analytical methods to study the levels of electrical activity of different muscle groups in natural and abnormal gait. Through direct analysis, we were able to quantify the intensity of electrical activity of each muscle at each stage of the gait cycle and found significant differences in muscle activity between the support and swing phases of different gait types. Then, the potential association between different gait types and muscle electrical activity was further revealed by principal component analysis of muscles with significant differences. In principal component analysis, we extracted the main components through dimensionality reduction processing of EMG data, which can effectively represent the electrical activity patterns of key muscles in the gait cycle. By contrast with direct analysis results, principal component analysis provides a more comprehensive and global perspective, revealing a more complex relationship between gait type and muscle activity.

In gait research, quantitative analysis of muscle activity patterns is important for identifying normal and abnormal gait. In this study, the analysis of IEMG data revealed the key roles of the left semitendinosus and the right biceps femoris in the whole gait cycle, and clarified the force generation characteristics of different muscle groups in various stages of gait, which provided quantitative reference for gait correction. Gait is influenced by a variety of factors, such as individual exercise experience, walking patterns, and the amplitude of joint movements, all of which can significantly affect the intensity of muscle electrical activity [24–25]. In natural gait, the integral EMG values clearly reflect the force generation characteristics of different muscles of an individual during walking. Statistical analysis of the integral EMG showed that the electrical activity levels of the medial left gastrocnemius, medial right gastrocnemius, left semitendinosus and lateral right gastrocnemius were significantly higher than those of the other muscles, suggesting that these muscle groups play an important role in the support and propulsion process. The results are consistent with the studies of Ping Huang and Winter, emphasising the dominant role of the medial gastrocnemius in the gait cycle [26–27]. The medial gastrocnemius showed the highest integral EMG values both in natural and abnormal gait, further validating its key force generation role in gait.

Based on the existing literature and gait analysis techniques, this study hypothesises that there are significant differences in the electrical activity characteristics of the gastrocnemius, hamstring and quadriceps muscles between natural and abnormal gait. These differences not only serve as indicators for recognising gait type, but also show different sensitivities at different stages of the gait cycle. The results verified the sensitivity of the gastrocnemius and biceps femoris muscles through the analysis of surface electromyographic data, especially in the different phases of the support phase versus the swing phase, which showed significant variations in their principal components.

Principal component analysis revealed significant differences between gait type and muscle electrical activity, particularly the key role of the left semitendinosus, right biceps femoris and medial left gastrocnemius in the gait cycle. The results showed that the left semitendinosus and the medial left gastrocnemius had significantly higher levels of electrical activity in gait type I than in gait type II, whereas the right biceps femoris had significantly higher levels of electrical activity in gait type II than in gait type I. The results showed that the left semitendinosus and the medial left gastrocnemius had significantly higher levels of electrical activity in gait type I than in gait type II. This suggests the key role of these muscles in knee flexion, hip extension and ankle stability, and provides theoretical support for individualised gait correction and rehabilitation training.

Specifically, the principal component analysis of the right biceps femoris muscle showed significant changes in the level of electrical activity during the weight-bearing period, the right single-support period, and the left single-support period. It was found that the level of electrical activity in the right biceps femoris muscle was significantly higher in Class II gait than in Class I gait during these periods, reflecting the fact that individuals with Class II gait have more intense muscle activity when the amplitude of hip and knee movements is greater [28]. In contrast, the principal component analysis of the left semitendinosus muscle showed that the knee extension of the left leg during striding was closely related to the muscle activity.

In addition, principal component analysis of the medial left gastrocnemius revealed changes in its electrical activity during the weight-bearing phase, the right single-support phase to the pre-pre-swing phase and the left single-support phase. This indicated that the activity strength and exertion level of the medial left gastrocnemius were significantly higher in class I gait than in class II gait. The stepping action of class I gait relies more on centrifugal control of the gastrocnemius muscle to ensure the stability and power output of the lower limb during propulsion [29]. In contrast, Class II gait relies more on the strength of other body parts to maintain forward momentum. If the weight-bearing capacity of the lower limbs is insufficient, or if body instability occurs, this process will be shortened, resulting in a rapid shift of the centre of gravity to the contralateral foot to maintain body balance.

Based on the existing gait analysis studies, this study provides an in-depth discussion on the characteristics of electromyographic activities of key muscle groups, especially the differences in electrical activities in natural and abnormal gait. By quantifying the changes in electrical activity of the gastrocnemius, hamstring and quadriceps muscles during different phases of gait, this study provides new data for gait analysis techniques and a theoretical basis for personalised rehabilitation training design.

Although this study revealed important findings in gait analysis, the limitations of the sample size and the singularity of the subjects’ age may limit the broad applicability of the findings. Future studies should further expand the sample size to include more types of individuals with gait abnormalities and incorporate more dimensional biomechanical parameters (e.g., joint moments and gait balance) to verify the reliability and generalisability of the present findings. In addition, the use of more complex statistical models, such as multivariate analysis or machine learning methods, may further improve the accuracy and application value of gait analysis.

## Conclusion

(1) There is a significant difference in the surface EMG activities of the left semitendinosus, right semitendinosus, right biceps femoris, and left gastrocnemius muscle medially and laterally between Class I gait and Class II gait in the swing phase and support phase. The higher level of EMG activity and the high contribution of muscles such as gastrocnemius, biceps femoris and semitendinosus in the Class I gait population indicate that these muscles can be used as the main indicators for normal gait recognition.
(2) When extracting the EMG time series curve function of lower limb movements, the screened left semitendinosus principal component IV and right biceps femoris principal component III can be used as sensitive parameter indicators of the type of lower limb movements in natural gait. The magnitude of their variations mainly occurred in the left and right single-support phase, suggesting that the single-support phase is a critical period for gait recognition, providing optimised parameters and technical means for quantitative evaluation of gait biomechanics.

## Supporting information

S1 Consent Form The informed consent form used to obtain participant consent. (DOCX)

S2 Dataset Raw data collected during the study. (ZIP)
